# Construction and validation of a risk prediction model for oral frailty in rural hypertensive patients

**DOI:** 10.3389/fpubh.2026.1687651

**Published:** 2026-02-05

**Authors:** Nina Feng, Yanzhen Tian, Shiji Zhang, Qiong Xiang, Shiyi Wei, Zhen Zhang, Jiajia Chen

**Affiliations:** 1 Zhuzhou Hospital Affiliated to Xiangya School of Medicine, Central South University, Zhuzhou, China; 2 Medical College of Jishou University, Jishou, China

**Keywords:** hypertension, old people, oral frailty, risk prediction, rural areas

## Abstract

**Objective:**

To explore the influencing factors of oral frailty in rural hypertensive patients in Zhuzhou City, Hunan Province, and construct a risk prediction model.

**Methods:**

A randomized sampling method was employed to investigate 538 older adults hypertensive patients in a rural area of Zhuzhou City, Hunan Province, using general data questionnaires, the Oral Hypertension Index-8 (OHI-8), and the EAT-10 scale. Among them, 377 cases were assigned to the modeling group, with internal validation conducted through 1,000 repeated Bootstrap sampling. An independent external validation group of 161 cases was also established.

**Results:**

The incidence of oral frailty in rural older adults hypertensive patients was 61.5%. Logistic regression analysis showed that age, education level, living alone, polypharmacy, smoking, dysphagia, xerostomia, and co-morbidities were influencing factors for oral frailty in rural older adults hypertensive patients (*P*<0.05). The modeling results showed an area under the curve of 0.781 (95%*CI*: 0.735–0.827). The Hosmer-Lemeshow goodness-of-fit test showed *χ*^2^ = 13.736, *p* = 0.089. The areas under the ROC curves for internal and external validation were 0.769 (95%*CI*: 0.754–0.779) and 0.810 (95%*CI*: 0.745–0.875), respectively, and the *p* values for the Hosmer-Lemeshow test were 0.089 and 1.133, respectively. The calibration plot showed a high degree of overlap between the predicted curve and the ideal curve, and the DCA plot indicated good applicability of the model in clinical decision-making.

**Conclusion:**

The incidence of oral frailty is high in rural older adults hypertensive patients. Its influencing factors include age, education, living alone, polypharmacy, smoking, dysphagia, xerostomia, and comorbidities. The oral frailty risk prediction model constructed based on these influencing factors has high predictive and discriminative power and can be used as a screening tool for the risk of oral frailty in rural older adults hypertensive patients.

## 1 Background

According to the China Cardiovascular Health and Disease Report (1), the prevalence of hypertension among adults aged ≥18 in China has reached 31.6%, with rural areas at 33.7%, significantly higher than urban areas (29.1%). The situation for hypertension prevention and control in rural areas is severe. In recent years, the association between hypertension and oral health has gradually attracted academic attention. Studies show that long-term hypertension can lead to oral microbiome imbalance and an increased risk of periodontal disease (2). Additionally, long-term vascular lesions and changes in the inflammatory microenvironment can impair oral mucosal repair capabilities, ultimately inducing oral frailty (3). Oral frailty describes a series of phenomena and processes such as declining oral function, reduced tooth count, and deteriorating oral hygiene, accompanied by a decreased interest in oral health management, leading to eating dysfunction and the deterioration of physical and cognitive functions (4). This is a further standardized explanation of poor oral health. When hypertensive patients experience oral frailty, it can trigger a series of serious consequences such as malnutrition, physical and psychological frailty, and social dysfunction. Their hospitalization rates and risk of falls increase, and it even raises the risk of death (5), forming a vicious cycle of “hypertension-oral frailty-overall health deterioration.”

Improving oral hygiene behaviors helps prevent future cardiovascular events, and oral hygiene management can improve adverse cardiovascular outcomes associated with poor oral health status (6). Surveys show that current older adults individuals hold incorrect oral health concepts such as “a toothache is not a disease” and “being toothless is normal.” These concepts delay the best treatment time for patients, leading to unsatisfactory oral hygiene, with 95.71% of older adults individuals having poor oral hygiene (7). A qualitative interview study in Australia showed that over 50% of cardiovascular disease patients had oral problems such as bad breath, gum recession, dental caries, and toothache, but paid insufficient attention to these issues and had a severe lack of understanding of the link between oral health and cardiovascular disease (8). Research in Iran also showed that although 75% of cardiovascular disease patients had some understanding of oral health, over 50% of them still exhibited poor oral hygiene behaviors (9). Research in the UK pointed out that 72.7% of cardiovascular stroke units had no specific management plans for patients’ oral conditions, and only 18.2% of units provided oral hygiene health education (10), highlighting the neglected state of oral health issues. Currently, oral health management in China mainly focuses on basic oral hygiene guidance and oral disease treatment. The research population rarely involves patients with oral frailty due to hypertension in rural areas, mostly focusing on urban older adults populations (11), diabetic patients (12), or cardiovascular disease patients (13). There is insufficient attention to oral frailty in rural patients and a lack of multi-dimensional oral frailty assessment tools. Coupled with the special geographical environment of rural areas, relatively scarce medical resources, and patients’ weak health awareness, the risk factors for oral frailty in patients are more complex.

Early identification and intervention for oral frailty are important means to reduce the occurrence of oral frailty in older adults hypertensive patients in rural areas. Risk prediction models can assess the risk of disease onset for individuals, predict the future risk of an individual developing a certain disease, screen high-risk groups, and provide targeted early interventions (14). This can provide more scientific decision-making tools for clinical intervention and healthcare professionals’ health education. In existing research, there are only oral frailty risk prediction models for community older adults (15), older adults patients with stroke (16), and older adults maintenance hemodialysis patients (17), lacking a risk prediction model for oral frailty in rural hypertensive patients. Therefore, this study’s oral frailty prediction model, constructed through a survey of oral frailty in older adults hypertensive patients in rural areas, serves as an important method for primary disease prevention and is of great significance for taking preventive measures to reduce the risk of oral frailty in older adults hypertensive patients in rural areas.

## 2 Objects and methods

### 2.1 Research subjects

In this study, using random sampling, 538 patients with hypertension in a rural area of Zhuzhou City, Hunan Province, were selected as the study subjects. Inclusion criteria: (1) Age > 60 years old and long-term residence in the area (residing for more than 6 months within 1 year); (2) patients who have been diagnosed with hypertension (18); (3) conscious and able to communicate normally. Exclusion criteria: (1) patients with diagnosed severe mental disorders, dementia, etc. who were unable to complete the questionnaire; (2) those with combined oral primary diseases; (3) those with combined severe heart, brain, kidney, and other organ insufficiency or those in the acute exacerbation of the disease. The logistic regression events per variable (EPV) method was used to calculate the sample size, i.e., a minimum of 10 positives were required for each predictor to be included in the prediction model (19). There are 21 influencing factors to be included in this study based on the literature review, combined with the literature to summarize the incidence of oral frailty as 71.6% (20), and considering the 10% loss of visit rate, the sample size is at least 327 cases, and 538 cases were finally included in this study, of which 377 cases were in the modeling group, and 161 cases were in the validation group. This study was approved by the Ethics Committee of Zhuzhou Central Hospital (Approval No.: 20233034-01). All participants provided informed consent.

### 2.2 Survey tools

#### 2.2.1 General information questionnaire

The research team developed it through discussion and included gender, age, education level, per capita monthly income, marital status, residence status, smoking history, drinking history, health insurance status, preferred tastes, sleep, xerostomia, frequency of daily brushing, frequency of visiting the dentist, and going out.

#### 2.2.2 Oral frailty index-8, OFI-8

Developed by Tanaka et al. (21), it was used to assess oral frailty in older adults, including five dimensions of denture use (1 item), swallowing ability (1 item), chewing ability (3 items), oral health-related behaviors (2 items), and social participation (1 item), with a total score of 0–11, and a score of ≥4 as oral frailty. Higher scores indicate poorer oral health, and each 1-point increase in score increases the risk of new oral frailty by 1.3 times. The overall Cronbach’s alpha coefficient for the scale was 0.692.

#### 2.2.3 Eating assessment tool-10, EAT-10

A screening tool developed by Belafsky et al. (22) in 2008 to assess dysphagia, the EAT-10 consists of 10 questions, each scored on a 4-point scale, with a score of 0 for no impairment, a score of 4 for severe impairment, and a total score of 3 or more is generally considered to be abnormal swallowing function. The overall Cronbach’s alpha coefficient for the scale was 0.947.

### 2.3 Research methods

In this study, data were collected face-to-face, and the investigator used consistent instructional language on-site to provide one-on-one guidance to each participant to ensure accurate completion of the questionnaire. After completion, the investigator checked the questionnaire on the spot and immediately added any missing or omitted items to ensure the completeness of the data. For patients with reading difficulties, a question-and-answer format was used to help patients complete the questionnaire to minimize completion errors due to personal factors. After the questionnaire is filled in, the researcher implements instant quality control, verifies the integrity of the questionnaire, and supplements the items with missing values on the spot. After double checking and confirming that there is no error, it is unifiedly taken back and placed. A total of 550 questionnaires were distributed in this study, and 538 valid questionnaires were recovered, with a recovery rate of 97.82%.

### 2.4 Statistical methods

SPSS 26.0 software and R4.3.3 were applied for statistical analysis. Normally distributed measurements were described by ( $\bar{x}$ ± s), and independent samples t-test was used for comparison of measurements between the two groups; quantitative information that did not conform to normal distribution was expressed by median and quartiles [M(P25, P75)], and rank sum test was used for comparison between groups; counting information was expressed by the number of cases and percentage (%), and *χ*^2^ test was used for comparison between groups. Variables that were statistically significant at *p* < 0.05 were included in multifactor logistic regression analyses, and the model fit was assessed by Hosmer-Lemeshow (H-L) goodness-of-fit by plotting column-line plots using R software. The area under the ROC and calibration curves was used to predict the differentiation and calibration of the model modeling group from the validation group. DCA was performed to analyze their clinical predictive value. Differences were considered statistically significant at *p* < 0.05.

## 3 Results

### 3.1 Incidence of oral frailty in rural hypertensive patients

A total of 538 patients were surveyed in the study, with 377 in the modeling group and 161 in the validation group. Two hundred and thirty two patients in the modeling group experienced OF, with an incidence rate of 61.5%. There were 162 males (43.0%) and 215 females (57.0%); the age range was 60–99 years.

### 3.2 Univariate analysis of oral frailty in rural hypertensive patients

The results of the univariate analysis showed that age, education level, living alone, economic status (monthly income), insurance type, polypharmacy, smoking, dysphagia, xerostomia, and comorbidities were associated with OF, and the differences were statistically significant (*p* < 0.05), as shown in Table 1.

**Table 1:** Univariate analysis of oral frailty in rural hypertension.

Variables	Category	Non-oral frailty (*n* = 145)	Oral frailty (*n* = 232)	*χ^2^*/*t* value	*P* value
Sex	Male	62 (42.8)	100 (43.1)	0.000^a^	1.000
	Female	83 (57.2)	132 (56.9)		
Age	—	70.91 (7.3)	74.11 (7.8)	−4.026^b^	<0.001
Education [*n*, (%)]	Primary school or below	32 (22.1)	101 (43.5)	21.993^a^	<0.001
	Junior high school	82 (56.6)	100 (43.1)		
	Senior high school or vocational school	21 (14.5)	27 (11.6)		
	Associate degree or above	10 (6.9)	4 (1.7)		
Marriage [*n*, (%)]	Married	111 (76.6)	175 (75.4)	0.015^a^	0.902
	Other	34 (23.4)	57 (24.6)		
Occupation before retirement [*n*, (%)]	Farmer	129 (89.0)	204 (87.9)	4.810^a^	0.440
	Worker	7 (4.8)	19 (8.2)		
	Teacher	1 (0.7)	3 (1.3)		
	Civil servant	2 (1.4)	3 (1.3)		
	Other	4 (2.8)	2 (0.9)		
Live alone [*n*, (%)]	Live alone	10 (6.9)	56 (24.1)	17.192^a^	<0.001
	Other	135 (93.1)	176 (75.9)		
Economic status (monthly income) [*n*, (%)]	<500	17 (11.7)	43 (18.5)	20.800^a^	0.001
	500–999	28 (19.3)	63 (27.2)		
	1,000–1,999	55 (37.9)	90 (38.8)		
	2,000–2,999	23 (15.9)	24 (10.3)		
	3,000–3,999	16 (11.0)	12 (5.2)		
	≥4,000	6 (4.1)	0 (0.0)		
Type of insurance [*n*, (%)]	None	2 (1.4)	14 (6.0)	8.126^a^	0.044
	New rural cooperative medical care/urban and rural residents’ medical insurance	135 (93.1)	211 (90.9)		
	Employees’ medical insurance	6 (4.1)	7 (3.0)		
	Commercial insurance	2 (1.4)	0 (0.0)		
Whether it is a special clinic for hypertension [*n*, (%)]	Yes	8 (5.5)	20 (8.6)	1.250^a^	0.264
	No	137 (94.5)	212 (91.4)		
Year of hypertension diagnosis	—	10 (5, 17)	10 (5, 16)	−0.093^c^	0.926
Have a family history of high blood pressure [*n*, (%)]	Yes	63 (43.4)	101 (43.5)	3.902^a^	0.142
	No	62 (42.8)	82 (35.3)		
	Unclear	20 (13.8)	49 (21.1)		
Polypharmacy [*n*, (%)]	≤4	132 (91.0)	164 (70.7)	20.705^a^	<0.001
	≥5	13 (9.0)	68 (29.3)		
Number of doses per day	—	1 (1, 1)	1 (0, 1)	−2.751^c^	0.006
Years on antihypertensive medication	—	8 (5, 15)	10 (5, 15)	−0.804^c^	0.421
Cigarette smoking [*n*, (%)]	Yes	29 (20.0)	90 (38.8)	13.732^a^	<0.001
	No	116 (80.0)	142 (61.2)		
Drinking wine [*n*, (%)]	Yes	28 (19.3)	83 (35.8)	10.866^a^	0.001
	No	117 (80.7)	149 (64.2)		
Favorite flavors [*n*, (%)]	Mild	11 (7.6)	40 (17.2)	11.241^a^	0.024
	Moderate	93 (64.1)	118 (50.9)		
	Salty	38 (26.2)	69 (29.7)		
	Sweet	1 (0.7)	0 (0.0)		
	Greasy	2 (1.4)	5 (2.2)		
Sleeping time per day [*n*, (%)]	≤6 h	85 (58.6)	129 (55.6)	0.331^a^	0.565
	>6 h	60 (41.4)	103 (44.4)		
Dysphagia [*n*, (%)]	Yes	97 (66.9)	191 (82.3)	10.942^a^	<0.001
	No	48 (33.1)	41 (17.7)		
Xerostomia [*n*, (%)]	Yes	64 (44.1)	137 (59.1)	7.386^a^	0.007
	No	81 (55.9)	95 (40.9)		
Reduced frequency of outings [*n*, (%)]	Yes	32 (22.1)	70 (30.2)	2.969^a^	0.085
	No	113 (77.9)	162 (69.8)		
Brush your teeth twice a day [*n*, (%)]	Yes	81 (55.9)	115 (49.6)	1.175^a^	0.278
	No	64 (44.1)	117 (50.4)		
Dentist visits once a year [*n*, (%)]	Yes	136 (93.8)	217 (93.5)	1.000^a^	1.003
	No	9 (6.2)	15 (6.5)		
Comorbidity [*n*, (%)]	Yes	90 (62.1)	93 (40.1)	16.394^a^	<0.001
	No	55 (37.9)	139 (59.9)		
Medication compliance [*n*, (%)]	Good compliance	111 (76.6)	178 (76.7)	0.578^a^	0.749
	Moderate compliance	16 (11.0)	24 (10.3)		
	Poor compliance	18 (12.4)	30 (12.9)		

a χ^2^value.

b *t* value.

c *Z* value.

### 3.3 Multifactorial analysis of oral frailty in rural older adults hypertensive patients

Oral frailty was used as the dependent variable (none = 0, yes = 1), and variables with statistically significant (*p* < 0.05) differences in the univariate analysis were included in the regression equation. The results showed that education level, living alone, economic status (monthly income), insurance type, polypharmacy, smoking, dysphagia, xerostomia, and comorbidities were the influencing factors of patients’ oral frailty (*p* < 0.05). See Tables 2, 3.

**Table 2:** Assignment of independent variables.

Variables	Coding
Education	Primary school or below (0, 0, 0, 0); Junior high school (0, 1, 0, 0); Senior high school or vocational school (0, 0, 1, 0); Associate degree or above (0, 0, 0, 1)
Live alone	Live alone = 1; Other = 0
Economic status (monthly income)	<500 (0, 0, 0, 0, 0, 0); 500–999 (0, 1, 0, 0, 0, 0); 1,000–1,999 (0, 0, 1, 0, 0, 0); 2,000–2,999 (0, 0, 0, 1, 0, 0); 3,000–3,999 (0, 0, 0, 0, 1, 0); ≥ 4,000 (0, 0, 0, 0, 0, 1)
Type of insurance	None (0, 0, 0, 0); New rural cooperative medical care / urban and rural residents’ medical insurance (0, 1, 0, 0); Employees’ medical insurance (0, 0, 1, 0); Commercial insurance (0, 0, 0, 1)
Polypharmacy	≥5 = 1; ≤ 4 = 0
Cigarette smoking	Yes = 1; No = 0
Dysphagia	Yes = 1; No = 0
Xerostomia	Yes = 1; No = 0
Comorbidity	Yes = 1; No = 0

**Table 3:** Logistic regression analysis of oral frailty in rural hypertension.

Variables	Category	*β*	SE	Wald χ^2^	*P*	OR	95%CI
Constant		−4.225	1.325	10.172	0.001	0.015	–
Age		0.045	0.017	6.754	0.009	1.046	1.012 ~ 1.082
Education	Junior high school	−0.817	0.286	8.182	0.004	0.442	0.250 ~ 0.768
	Senior high school or vocational school	−0.783	0.409	3.667	0.055	0.457	0.204 ~ 1.019
	Associate degree or above	−2.638	0.765	11.885	<0.001	0.071	0.014 ~ 0.299
Live alone	Yes	1.155	0.432	7.137	0.008	3.173	1.414 ~ 7.825
Polypharmacy	≥5	1.253	0.396	9.995	0.002	3.500	1.666 ~ 7.981
Cigarette smoking	Yes	0.633	0.317	3.971	0.046	1.882	1.016 ~ 3.542
Dysphagia	Yes	0.656	0.291	5.062	0.025	1.926	1.091 ~ 3.428
Xerostomia	Yes	0.748	0.250	8.977	0.003	2.112	1.300 ~ 3.464
Comorbidity	Yes	0.613	0.252	5.911	0.015	1.846	1.128 ~ 3.036

### 3.4 Construction and internal validation of a predictive model for risk of oral frailty in rural hypertensive patients

**Figure 1:**
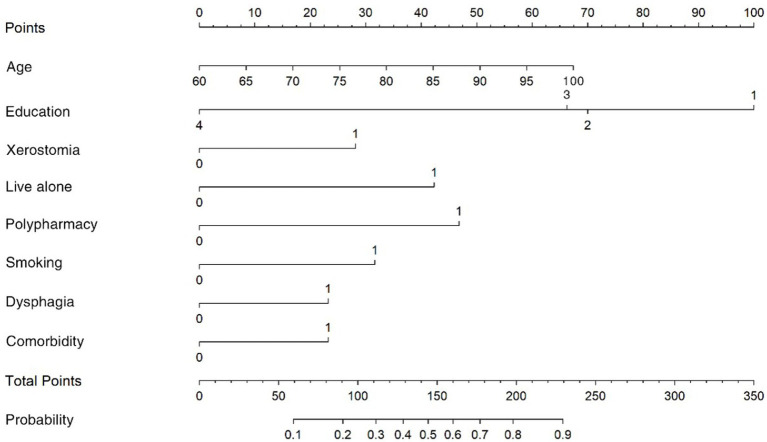
Nomogram for predicting the risk of oral frailty in rural hypertensive patients.

**Figure 2:**
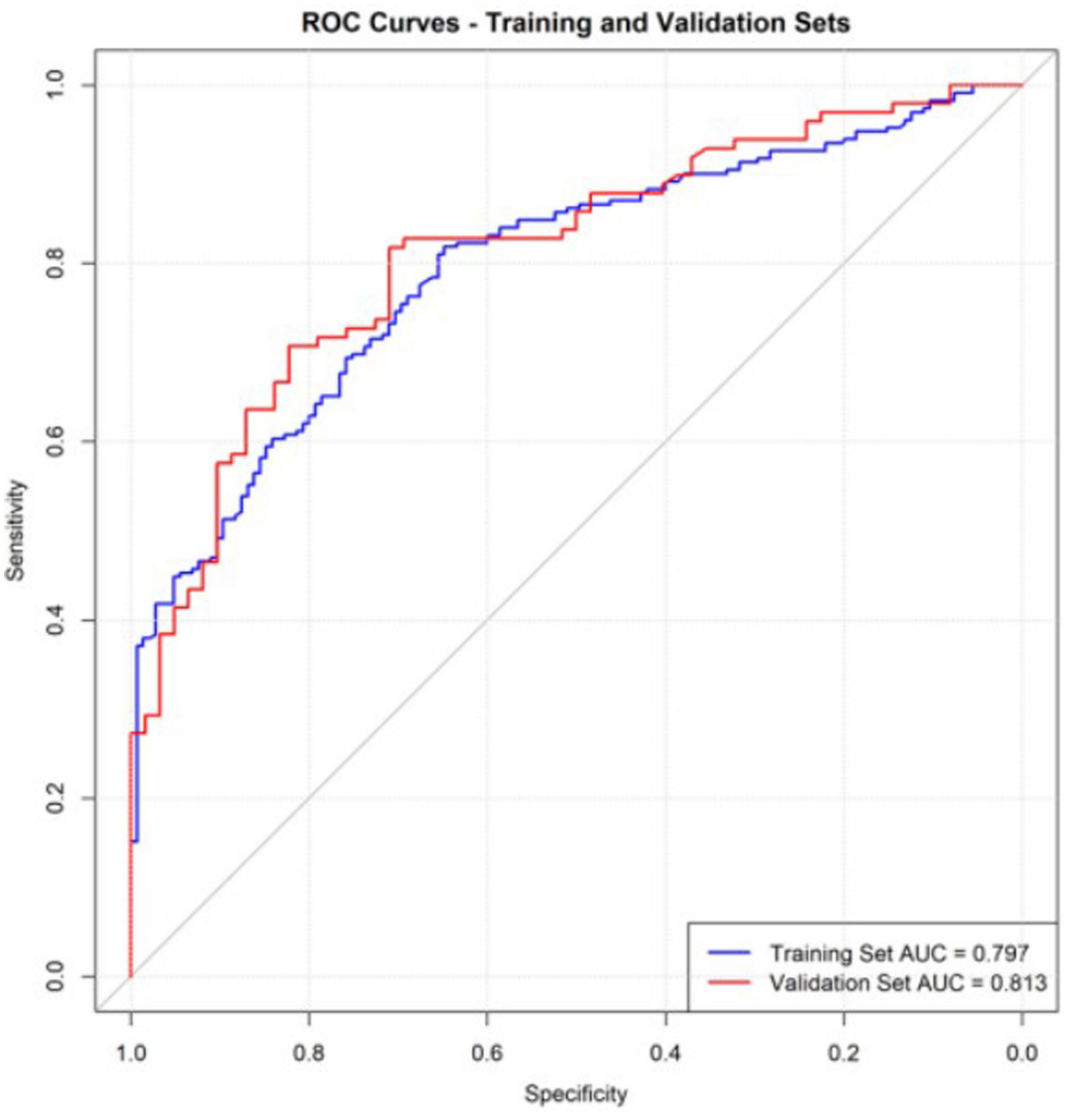
The receiver operating characteristic curve for oral frailty prediction model.

**Figure 3:**
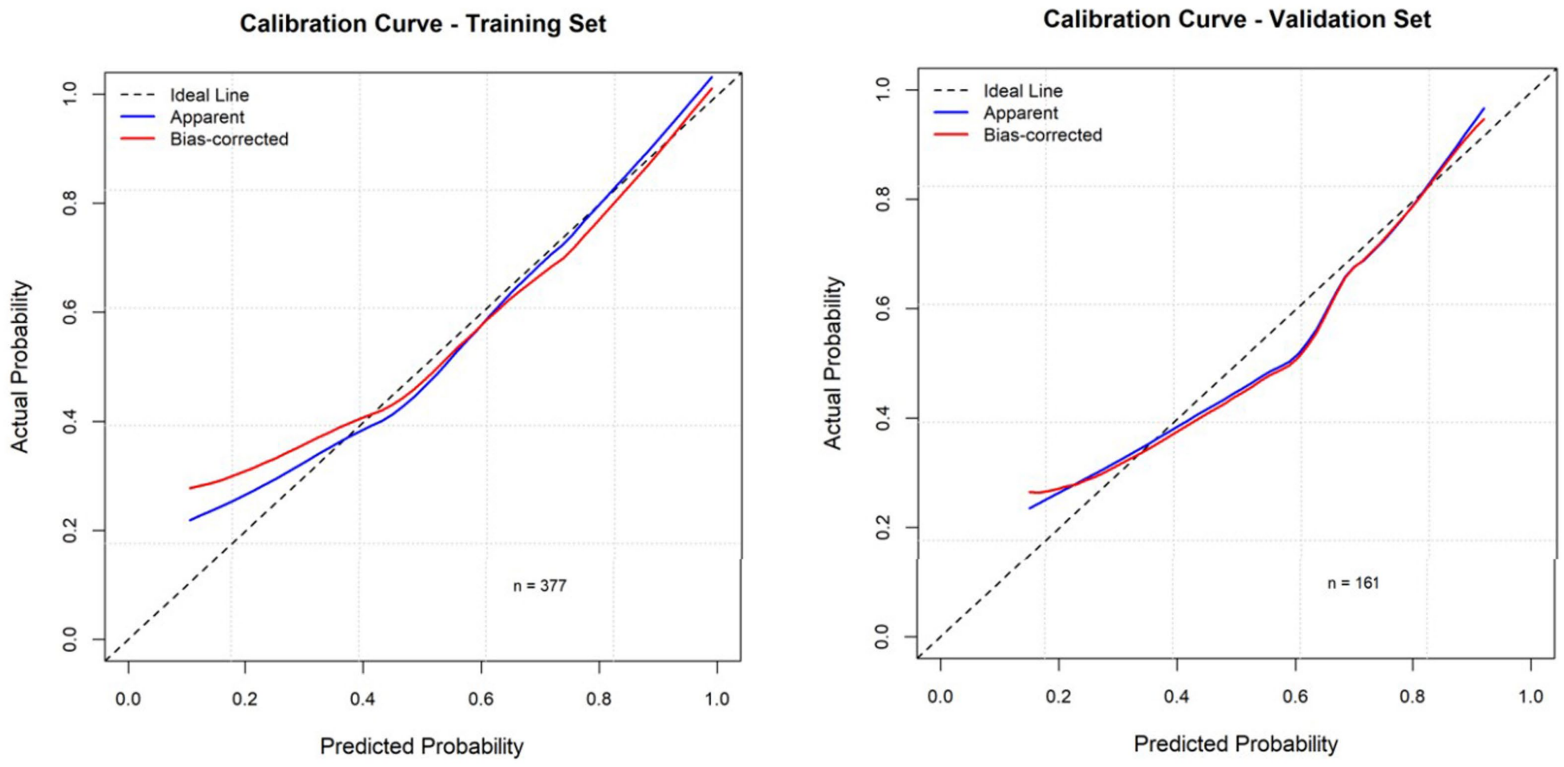
The calibration curve of model internal validation (training set and validation set).

Based on the results of the multivariate analysis of oral frailty in rural hypertensive patients, the partial regression coefficients of each variable were obtained to construct a risk prediction model for oral frailty in rural older adults hypertensive patients. The final formula is: Logit(P) = −4.225 + 0.045 × Age − 0.817 × Junior High School − 0.783 × High School or Technical Secondary School − 2.638 × College and above + 1.155 × Living Alone + 1.253 × Polypharmacy ≥ 5 types + 0.633 × Smoking + 0.656 × Dysphagia + 0.748 × Xerostomia + 0.613 × Comorbidity. A nomogram prediction model was constructed using R4.3.2. Each variable corresponds to a score on the nomogram score axis with a perpendicular line. The sum of the scores of all variables corresponds to the numerical value of the probability of oral frailty, which is the predicted value of the risk of oral frailty. A higher total score indicates a higher risk of oral frailty in rural hypertensive patients, as shown in Figure 1. The area under the ROC curve of the prediction model was 0.781 (95%*CI*: 0.735–0.827), as shown in Figure 2. The maximum Youden index was used as the optimal cutoff value for the prediction model. The Youden index was 0.467, with a sensitivity of 0.819 and a specificity of 0.648. The Hosmer-Lemeshow goodness-of-fit test showed *χ*^2^ = 13.736, *p* = 0.089, as shown in Figure 3, indicating that there was no statistically significant difference between the internally predicted probability of oral frailty in rural older adults hypertensive patients and the actual probability, suggesting a good fit and high predictive value of the model. Analysis of 1,000 bootstrap resamples of the modeling group data showed that the area under the ROC curve of the model was 0.769 (95%*CI*, 0.754–0.779), further indicating that the model has good predictive value, as shown in Figure 2. In the calibration plot, the calibration curve showed a high degree of overlap with the ideal curve, indicating good calibration of the model and high similarity between the observed and predicted values, as shown in Figure 3.

### 3.5 External validation of a predictive model for oral frailty risk in rural hypertensive patients

**Figure 4:**
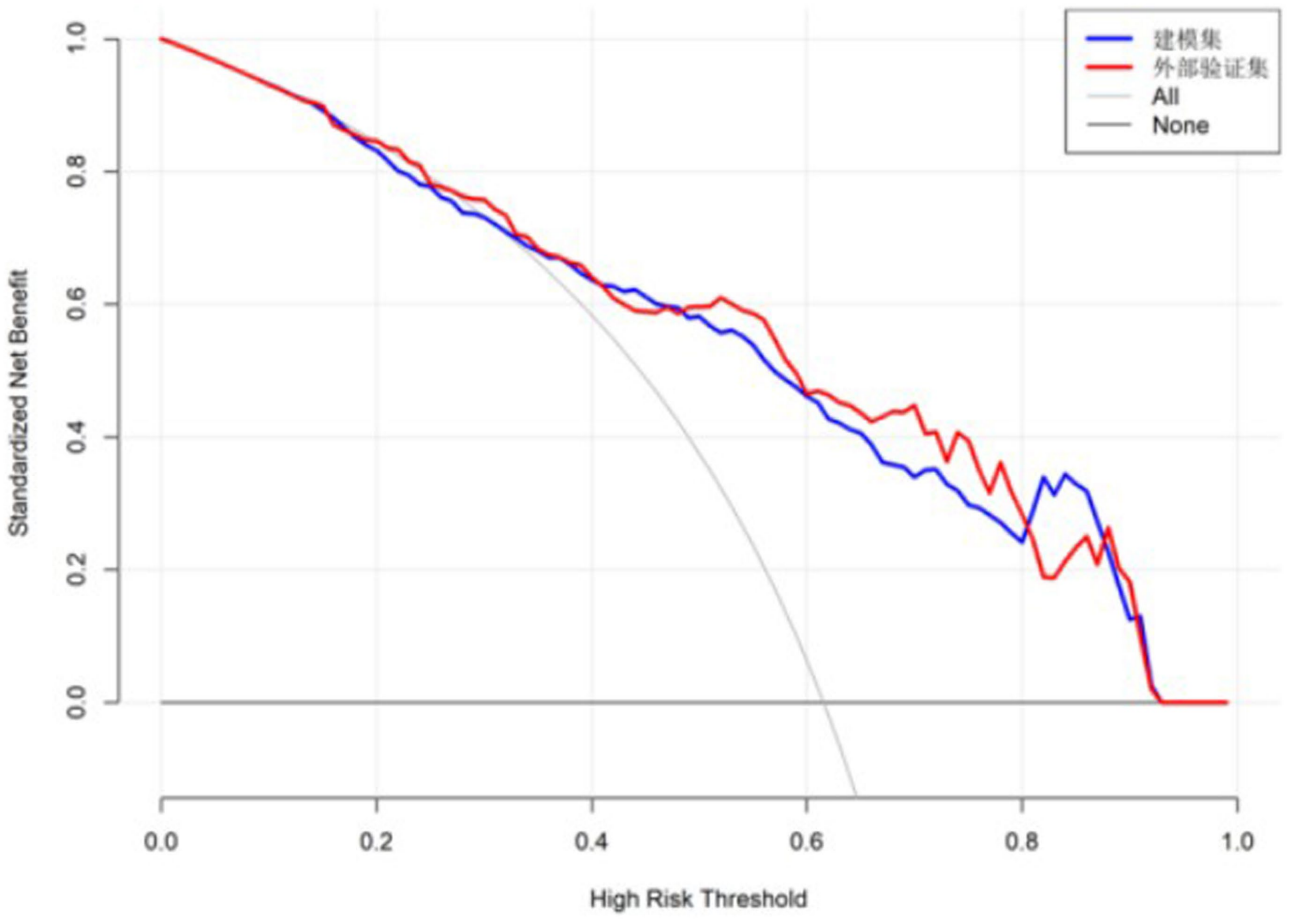
The decision curve of the model (training set and validation set).

External validation of the model was performed using validation set data. The external validation results showed that the area under the ROC curve of the risk prediction model was 0.813 (95% *CI*: 0.748–0.879), as shown in Figure 2. The calibration curve and the ideal curve in the calibration plot showed a high degree of agreement. The Hosmer-Lemeshow goodness-of-fit test showed χ^2^ = 12.444, *p* = 0.447, indicating that the model has good predictive ability, as shown in Figure 3. The DCA plot showed that at different risk thresholds, the net benefit of this model was higher than the “no intervention” and “all intervention” strategies, indicating that the model has good applicability in clinical decision-making, as shown in Figure 4.

## 4 Discussion

### 4.1 Current status of oral frailty in rural hypertensive patients

The results of this study show that the incidence of oral frailty among rural hypertensive patients is as high as 61.5%, which is higher than previous studies on communities (11). This may be due to the relatively backward economic development in rural areas, traffic barriers, limited medical resources, and lack of professional oral medical institutions, so patients with oral problems cannot receive regular treatment (23). Furthermore, patients in rural areas often lack awareness of the importance of oral health and do not recognize its close connection to overall health. Additionally, hypertension may contribute to oral diseases such as periodontitis through mechanisms including vascular damage, inflammatory responses, medication side effects, and immune function alterations, thereby increasing the risk of oral frailty (24). Moreover, suboptimal chronic disease management in rural areas accelerates disease progression and development. In addition, chronic disease management in rural areas is weak, which accelerates the occurrence and development of diseases. Therefore, attention should be paid to the oral health of rural hypertensive patients, and targeted screening, assessment, and intervention should be actively carried out, especially in remote rural areas, to improve the quality of life related to oral health and to extend basic screening, education, and simple interventions to the village or home level.

### 4.2 Risk factor interactions in the prediction model for oral frailty in rural hypertensive patients, and corresponding preventive measures can be formulated based on the identified risk factors

The results of this study indicate that “age” and “dysphagia” are risk factors for oral frailty, with older hypertensive patients facing a higher risk of developing oral frailty, which is consistent with findings from other studies (25). On the one hand, this may be due to the decrease in alkaline phosphatase activity, regenerative capacity, and osteogenic activity of periodontal ligament cells with increasing age, leading to physiological gingival atrophy and demineralization and softening of cementum, which can cause diseases such as periodontitis and dental caries, leading to the occurrence of oral frailty (26). On the other hand, increasing age can lead to a decline in the function of nerves, muscles, and salivary glands in the oral pharynx, resulting in chewing difficulties and dysphagia. Dysphagia is an important sign of oral frailty, often accompanied by a decline in chewing function. Food cannot be fully chewed and mixed, affecting the self-cleaning ability of the oral cavity (27), and increasing the risk of oral diseases. At the same time, patients with dysphagia may experience discomforts such as difficulty swallowing, choking, and coughing. Coupled with the impact of hypertension, this directly restricts the patient’s choice of food types (28), easily leading to malnutrition, weakening the repair ability and immunity of oral tissues, and accelerating the development of oral frailty (29). The results of this study show a high incidence of dysphagia (76.4%) in rural hypertensive patients, suggesting that older adults patients with dysphagia should be a key focus. Therefore, it is recommended to establish a priority screening mechanism for the older adults, increase health follow-ups for older adults, and appropriately include oral function screening in rural follow-up visits. For older adults hypertensive patients with dysphagia, simple home rehabilitation training and food texture guidance should be provided, along with basic cleaning and treatment services, and home denture maintenance.

This study’s results indicate that “living alone,” “comorbidities,” “polypharmacy,” and “xerostomia” are risk factors for oral frailty. Living alone is common in rural areas, and the incidence of oral frailty among rural hypertensive patients living alone in this study reached 31.4%. Living alone may increase the risk of oral frailty through multiple pathways. On one hand, patients living alone have limited communication with the outside world and reduced daily social activities, leading to insufficient activity of the muscles around the mouth and pharynx, which may result in slowed tongue movement, reduced chewing ability, and weakened tongue pressure (30). On the other hand, a lack of social interaction can induce negative emotions such as loneliness and depression. These psychological factors can increase the risk of oral diseases by affecting saliva secretion and oral self-cleaning ability (29). Furthermore, patients living alone often have weak social support networks and lower self-management efficacy, leading to poor blood pressure control and neglect of oral health. Research indicates that the presence of caregivers can help older adults maintain good oral hygiene through daily interactions and support, thereby reducing the risk of oral diseases (31). This study found that the incidence of oral frailty in rural patients with comorbidities (76.3%) was significantly higher than in those without comorbidities (48.9%). Comorbidities not only increase the overall disease burden but also weaken the defense capabilities of oral tissues, thereby increasing susceptibility to oral frailty (16). For example, stroke patients, due to neurological damage, experience a decline in oral self-management ability, increasing the risk of oral diseases (32). Polypharmacy is a common consequence of the above situations. In this study, 30% of hypertensive patients took ≥5 medications, indicating that polypharmacy is relatively common among rural hypertensive populations, consistent with the findings of Hironaka et al. (30). Among these, long-term use of antihypertensive drugs, statins, etc., can inhibit saliva secretion or alter the state of the oral mucosa, leading to xerostomia. Xerostomia (33), by weakening the lubricating, buffering, and antibacterial functions of saliva, promotes the accumulation of oral plaque, caries, and periodontal disease (34), and these oral diseases further promote the decline of oral function. Therefore, we recommend targeted interventions in rural areas, suggesting that village doctors regularly visit older adults individuals living alone and left-behind older adults, focusing on supervising their oral hygiene, medication use, and chronic disease management, and promptly identifying issues such as xerostomia, difficulty swallowing, or chewing. For those on polypharmacy, regular medication reviews should be conducted, with township doctors collaborating with county-level pharmacists or remote experts to assess medication use and adjust medications while ensuring chronic disease control.

The results of this study show that “education level” and “smoking” are risk factors for oral frailty. The present survey revealed that the prevalence of oral frailty among rural hypertensive patients increased with lower educational levels (with OR values of 0.398, 0.494, and 0.224, respectively), which aligns with findings from other studies (20). Compared with urban or community populations, rural residents generally have lower education levels (35) and weaker oral health literacy. In this study, only 39.5% of respondents reported brushing their teeth twice a day, lacking basic knowledge of oral health, and having insufficient awareness of the importance of regular oral examinations. Patients with lower education levels also have poorer self-management abilities, manifested as lower blood pressure control rates (36), and are more prone to unhealthy lifestyle habits such as smoking and drinking, which exacerbate the impact on oral health. In this study, the prevalence of oral frailty among rural hypertensive patients who smoked (36.3%) was significantly higher than that among non-smokers (18.4%), which is consistent with previous research findings (37). The harm of smoking to hypertensive patients is multifaceted. Smoking not only stimulates their sympathetic nerves, causing vasoconstriction and spasm, leading to increased peripheral vascular resistance, exacerbating blood pressure fluctuations, and making blood pressure more difficult to control (38). It also promotes the deposition of dental calculus, inhibits the activation and proliferation of immune cells, and weakens periodontal immune defense due to harmful components such as tar and nicotine in tobacco (39). Secondly, the high temperature and chemical irritants produced by smoking can damage the integrity of the oral mucosal epithelium, leading to oral mucosal lesions and altering the balance of oral microorganisms (40), thereby significantly increasing the risk of oral frailty in hypertensive patients. In the study, the proportion of drinking patients with oral frailty reached 33.2%, which was significantly higher than that of non-drinking patients (20.8%). Alcohol stimulates the oral mucosa, leading to a decrease in mucosal barrier function, and also damages neutrophils, weakening the body’s immune defense (41). The sugars and acidic substances in alcohol can also damage teeth, leading to oral problems such as periodontal disease and dental caries (42). Drinking may also increase the pressure on the blood vessel walls of hypertensive patients, reduce the efficacy of antihypertensive drugs, and increase the risk of complications (43), indirectly promoting the occurrence of oral frailty. Although drinking was not included in the multivariate model of this study, its association with adverse oral outcomes deserves continued attention and intervention in rural populations. Therefore, it is particularly important to popularize oral health knowledge and self-management skills, and promote correct brushing and regular oral examinations among groups with lower education levels. It is recommended to routinely inquire about smoking and drinking behaviors during chronic disease follow-ups in rural areas, carry out smoking cessation/alcohol restriction interventions, and incorporate smoking cessation into grassroots medical services.

In summary, oral frailty in rural hypertensive patients is associated with age, dysphagia, living alone, co-morbidities, polypharmacy, xerostomia, education level, and smoking. These factors are intertwined, forming a vicious cycle that exacerbates the risk of oral frailty in rural hypertensive patients. Therefore, it is essential to comprehensively consider the aforementioned risk factors and, based on the established risk prediction model, accurately identify high-risk individuals to implement targeted prevention and intervention measures early. At the same time, during the investigation, our research team found that when rural family doctors conduct home visits, their focus is primarily on the management of chronic diseases such as hypertension, neglecting patients’ oral health problems. This leads to the omission of oral problems in hypertensive patients at the initial stage of primary healthcare, delaying the timing of oral health guidance and intervention. Therefore, primary care will play a crucial role in this process and needs to strengthen primary oral management. It is recommended that primary healthcare institutions conduct training for village doctors and regularly organize village doctors for home visits. They can also provide various forms of services, such as centralized health lectures, to offer guidance on hypertension self-management, detailed explanations of medication-related knowledge, oral health-related knowledge, and popular education on healthy lifestyles, with the aim of reducing the risk of oral frailty.

### 4.3 The risk prediction model for oral frailty in rural hypertensive patients has good predictive efficacy

The area under the ROC curve for the training set in this study was 0.781, indicating that the model has a good ability to predict whether rural hypertensive patients will develop oral frailty. The calibration plot of the risk prediction model showed a high degree of overlap between the calibration curve and the ideal curve, and the Hosmer-Lemeshow goodness-of-fit test showed *p* = 0.089, suggesting high accuracy in the model’s prediction results. The areas under the ROC curves for internal and external validation in this study were 0.769 and 0.831, respectively, further indicating that the model has good predictive value. Therefore, the risk prediction model for oral frailty in rural hypertensive patients constructed in this study has high predictive accuracy, which can provide a powerful tool for clinical medical staff to early identify and manage the risk of oral frailty in rural hypertensive patients, and lay the foundation for the formulation of intervention measures.

## 5 Summary

The incidence of oral frailty in rural hypertensive patients is high. The further constructed risk prediction model of oral frailty in rural hypertensive patients has good predictive efficacy, which is conducive to the early identification of oral frailty in rural hypertensive patients by medical staff, and to carry out assessment, screening, intervention and other activities for rural high-risk groups, so as to prevent or delay the occurrence of oral frailty and improve the oral health level of rural hypertensive patients. This study has not carried out a large-scale study in rural areas. In the future, we can further expand the research area, carry out multi-center, large-sample and prospective studies, and deeply understand the situation of oral frailty in rural areas. As a preliminary screening tool, this study includes limited variables. However, oral frailty is affected by multi-dimensional factors. It is recommended to include more relevant variables in the future, further improve the prediction model, and formulate relevant intervention measures to effectively deal with the occurrence of oral frailty in rural hypertensive patients.

## Data Availability

The original contributions presented in the study are included in the article/supplementary material, further inquiries can be directed to the corresponding author.
